# Pakistan’s First Child & Adolescent Psychiatry Inpatient Unit: Characteristics of admitted patients and response to treatment over a 7-year period

**DOI:** 10.12669/pjms.37.2.2611

**Published:** 2021

**Authors:** Nazish Imran, Zubair Hassan Bodla, Aftab Asif, Rabia Shoukat, M. Waqar Azeem

**Affiliations:** 1 Prof. Dr. Nazish Imran, MBBS, FRCPsych (London), MRCPsych (London), MHPE, Department of Child & Family Psychiatry, King Edward Medical University, Mayo Hospital, Lahore, Pakistan; 2 Dr. Zubair Hassan Bodla, MBBS, Medical Officer, Department of Child and Family Psychiatry, King Edward Medical University, Mayo Hospital, Lahore, Pakistan; 3 Prof. Aftab Asif, MRCPsych (London), Professor & Head, Academic Department of Psychiatry & Behavioural Sciences, King Edward Medical University, Mayo Hospital, Lahore, Pakistan; 4 Rabia Shoukat, MS Psychology. Intern Psychologist, Department of Child & Family Psychiatry, King Edward Medical University, Mayo Hospital, Lahore, Pakistan; 5 Prof. Dr. M. Waqar Azeem, MD, DFAACAP, DFAPA, Chair, Department of Psychiatry / Child & Adolescent Psychiatry, Sidra Medical and Research Center, Professor of Psychiatry, Weil Cornell Medicine, Doha Qatar

**Keywords:** Adolescent, Child, Inpatient, Psychiatry, Psychiatric services

## Abstract

**Background & Objective:**

Child & adolescent mental health needs to be considered as an integral component of overall health, however significant gaps exist in service provision especially inpatient services in Pakistan. The paper presents the characteristics of admitted youths and response to treatment in Pakistan’s first dedicated child & adolescent psychiatry inpatient unit in Lahore over a period of first seven years. The aim of this study was to better understand the various characteristics of children and youth admitted to this inpatient unit and response to treatment over a seven years’ period since the inception of the unit.

**Methods:**

Inpatient medical records of children & adolescents admitted to dedicated Child & Adolescent Inpatient Unit at King Edward Medical University, Lahore were reviewed. Data was extracted regarding referral patterns, sociodemographic factors and diagnosis for the first seven years, from 2012 to 2019. Patients’ scores on Strengths and Difficulties Questionnaire and Clinical Global Impressions Scales administered during intake were also reviewed.

**Results:**

Six hundred and thirty-four (634) patients, 56% (355) being females were admitted to the unit during seven years with mean age of 12.3 ± 2.3. Mean duration of admission was 15.60 ± 6.3 days. Most predominant ICD-10 Axis-I psychiatric diagnosis were neurotic, stress related and somatoform disorders (262); mood disorders (78); schizophrenia, schizotypal & delusional disorders (77) and behavioral and emotional disorders with onset usually occurring in childhood and adolescence (44). One hundred and fifty-nine (25%) children had comorbid diagnosis of intellectual disability on Axis-III. Strengths and difficulties questionnaire scores were in abnormal range for significant proportion (>50 %) of patients. CGI mean scores showed marked improvement at discharge.

**Conclusion:**

Neurotic, stress related and somatoform disorders are the most common diagnosis in youth needing inpatient treatment in Pakistani setup. Study results indicate that there is a clear need for specialized inpatient child and adolescent services such as ours in low- & middle-income countries.

## INTRODUCTION

Low income countries like Pakistan face a multitude of social adversities including poverty, malnutrition, rapid urbanization, educational deprivation, drug abuse, increased crime, terrorism etc. thus increasing the risk of mental health problems in youth. Despite high prevalence of psychiatric problems in young people^1^,^2^, child mental health services in Pakistan are still in their infancy. Although increased attention is now being paid to inadequacies of child mental health provision recently, there are only few dedicated facilities providing these services. They are all located in urban centers and provide services in outpatient settings. However; some young people have severe and complex problems that require inpatient assessment and treatment. Most of these patients end up being placed in adult general psychiatric units, far from an ideal scenario. Young children presentation of psychiatric problems differs significantly from adults and they are also vulnerable to exploitation by adults and may become extremely distressed with disturbed adult behaviors. Because of these factors and need for young people to be treated in an environment appropriate to their age and developmental stage, Pakistan’s first specialist inpatient child and adolescent psychiatric unit was established within the public sector at the King Edward Medical University/ Mayo Hospital Lahore with six beds in 2012 (AACAP Newsletter Jan-Feb 2014). It has dedicated staff including consultant child psychiatrist, clinical psychologists, play therapist, speech therapist and trained nurses and other support staff. The unit provide comprehensive assessments in close collaborations with families and schools. Various treatment modalities being used in inpatient unit include psychotropic medications, family therapy, individual therapy, parenting work, play therapy, and help in developing individualized education program. An evaluation of current state of services, population of patients utilizing the inpatient services will help with future direction for service expansion and services utilization.

## METHODS

The records of all children and adolescents admitted to Child & Family Psychiatry Department Inpatient Unit in KEMU/ Mayo Hospital Lahore for a period of seven years since its inception (October 2012- December 2019) were reviewed. Deidentification of medical records was ensured by removing the first page of the patient file having patient name, date of birth, address, contact number and any other identifiable information in records, instead a simple identification number was used for files and data management. A proforma was designed to assist in extracting information from the case notes. Proforma included demographic information (age, gender, source of referral), duration of admission and diagnosis on five axes of ICD-10. Furthermore, scores on routinely administered assessment tools for admitted patients, i.e. strengths and difficulties questionnaire (SDQ) and clinical global Impression Scale (CGI) [severity on admission & discharge, improvement and efficacy index subscales] were analyzed.

### Strengths and Difficulties Questionnaire (SDQ)

All the children and adolescents requiring admission in the department were screened at the time of admission for behavioral and psychological problems by using a universally validated screening tool, Strengths and Difficulties Questionnaire (SDQ),completed by the caregivers.^3^It has an Urdu version, which has been validated to assess psychopathology in children in Pakistan.^4^,^5^SDQ has subscales of conduct, hyperactivity, emotional, peer problems and prosocial behavior. All scales excluding the last are added to generate a total difficulties score (0-40). Total difficulties and categories scores can be coded in normal, borderline and abnormal categories.

### Clinical Global Impression Scale (CGI)

CGI is a brief clinician rated instrument that takes into account history, psychosocial circumstances, symptoms, behavior and impact of symptoms on patient daily functioning. It is concise and easy to administer. It is one of the most widely used tools in Psychiatry.^6^ CGI consists of three different global measures.

### Severity of illness (CGI-S)

overall assessment of the current severity of the patient’s symptoms on scale of 1 (normal, not ill) -7(among the most extremely ill patients). The CGI-S was rated at admission (CGI-S_adm_) and at discharge (CGI-S_dis_);

### Global improvement

overall comparison of the patient’s baseline condition with his current state on seven-point scale (CGI-I). CGI-I) is rated from 1 (very much improved) to 7 (very much worse). The CGI-I was rated at discharge only;

### Efficacy index

overall comparison of the patient’s baseline condition to a ratio of current therapeutic benefit and severity of side effects (CGI-E). The Clinical Global Impression – Efficacy Index is a 4 point × 4-point rating scale that assesses the therapeutic effect of the treatment as 1. unchanged to worse; 2. minimal; 3. moderate; 4. marked; and Side effects rated as 1. None;2. do not significantly interfere with patient’s functioning;3. significantly interferes with patient’s functioning;4. outweighs therapeutic effect.

Patients for whom there was not enough data to comprehensively assess a patient (Missing data / SDQ &CGI not completed) as well as those who had a very brief length of stay (less than <2 days, either patient left against medical advice or needed to be transferred to another unit) were excluded from the study. This was done because initial few days may be very unsettling for children and little observation or assessment can be completed in such short time period. The data was collected in a deidentified format and data collection involved no direct contact with patients or their families. Data was analyzed using SPSS version 20. Descriptive statistics were calculated.

## RESULTS

Overall, six hundred and thirty-four (634) patients were admitted in the unit during first seven years with mean age of 12.3 ± 2.3. Median age was 13 years. Majority were females (355; 56%) and most were brought to hospital by their family (424; 67%). Other major referral sources included emergency department, pediatrics, neurology, plastic surgery and family physicians. Mean duration of admission was 15.60 ± 6.3 days. Almost all patient’s families had monthly income of less than 30,000 PKR (equivalent to approximately £189-$200).

### Diagnosis

Most predominant ICD-10 Axis I psychiatric diagnosis were neurotic, stress related and somatoform disorders (262; 41.32%); mood disorders (78; 12.30%); schizophrenia, schizotypal & delusional disorders (77; 12.14%) and behavioral and emotional disorders with onset usually occurring in childhood and adolescence (44; 6.94%) (Table-I) One hundred and fifty nine children had comorbid diagnosis of intellectual disability on Axis-III. Axis IV (medical comorbidities) & Axis-V (abnormal associated psychosocial situation) were noted in 50 & 109 of admitted children respectively.

**Table-I:** ICD-10 Axis I, III, IV and V Diagnosis in Inpatients Sample. (n=634)*

ICD-10 Code	Diagnostic Categories	No (%) of patients
Axis I:	Clinical Psychiatric Disorders	
F20-29	Schizophrenia, Schizotypal and Delusional Disorder	77(12.14%)
	Acute and Transient Psychotic Disorder	51
	Schizophrenia	26
F30-39	Mood [affective]Disorder	78 (12.30%)
	Depressive episode	50 (6 with psychotic symptoms)
	Bipolar Affective Disorder	28
F40-48	Neurotic, Stress-related and Somatoform Disorders	262 (41.32%)
	Dissociative[conversion]Disorders	185
	Somatoform Disorders	39
	Generalized Anxiety Disorder	10
	Obsessive-Compulsive disorder	5
	Reaction to Severe Stress, and Adjustment disorders	
	Acute Stress Reaction	17
	Post-Traumatic Stress Disorder	6
F90- F98	Behavioral and Emotional Disorders with Onset usually occurring in Childhood and Adolescence	44 (6.94%)
	Conduct Disorders	24
	Oppositional Defiant Disorder	13
	Hyperkinetic disorder	2
	Tic disorder	1
	ADHD	3
	Autism	1
	Miscellaneous:	2 (0.31%)
	Drug induced Side effects	2
Axis III. **	Intellectual Disability (ID)	
F70-79	Intellectual Disability (Mental Retardation in ICD-10)	159 (25.07%)
Axis IV:	Medical Illness	50(7.88%)
	Epilepsy	40
	Tuberculosis	3
	Cervical radiculopathy	1
	Chronic Bronchitis	1
	Drug reaction	2
	Facial Palsy	1
	Rickets	1
	Wilson’s disease	1
Axis V:	Psychosocial factors	109(17.19%)
	Abnormal interfamilial relationships	50
	Mental disorder in child primary support group.	19
	Abnormal qualities of upbringing.	1
	Acute Life events	
	-Extra familial sexual abuse	10
	-Chronic interpersonal stress associated with school/work.	29

* Two patients’ data on diagnosis was missing/unclear, because of either very short admission/ incomplete evaluation. (Patient either left against medical advice or needed to be shifted to other units in the hospital before comprehensive evaluation was completed).

** Axis II diagnosis was not given due to unavailability of formal assessment tools for specific learning disorders like reading and mathematics disorders.

### IRB Approval

Not needed being a inpatient records review.

### Strengths and Difficulties Questionnaire. (SDQ)

A large proportion of admitted children had strengths and difficulties questionnaire scores in abnormal range particularly in emotional and peer problems subcategories (Table-II).

**Table-II:** Strengths and Difficulties Questionnaire Borderline-Abnormal Categories Scores in the Inpatient Sample. (n=598 due to missing data).

SDQ	Borderline-Abnormal Range	

	N	(%)
Total Score	340	56.8
Emotional	340	56.8
Peer problems	327	54.6
Conduct	296	49.4
Hyperactive	188	31.4
Prosocial	135	22.5

### Clinical Global Impression (CGI)

Significant improvement was seen at discharge in patients with Mean Clinical Global Impression (CGI) severity score on admission being 4.4 ±1.0 (Markedly ill) and on discharge being 1.9 ±0.9 (Borderline mentally ill). A Wilcoxin Signed-Rank test showed that admission in inpatient unit did result in significant change in Mean clinical Global Impression severity score (Z=-9.97; P=0.000). CGI Improvement mean score was 1.7 ± 0.7 (Marked improvement). CGI-Efficacy index was 1.6 ±1.6 (Marked improvement with side effects of pharmacotherapy that do not significantly interfere with patient’s functioning).

## DISCUSSION

This is a unique study and first attempt to provide a snapshot of the provision and activities of the first dedicated child and adolescent in-patient unit in Pakistan. Knowing that an effective child and adolescent mental health facility can be established in a low-income country is important given a global trend towards increasing admissions into child and adolescent units.^7^Compared with developed countries, child psychiatric services in Pakistan are underdeveloped, both in quantity and quality of human and infrastructure resources mainly due to inadequate healthcare resources allocation to psychiatric services and shortage of trained staff. Despite high prevalence of child psychiatric disorders, only a limited number of these vulnerable children receive any psychiatric services. Various barriers including stigma, economic adversity, socio-cultural factors and parental & young people perceptions regarding mental health are key factors in underutilization of mental health services.^8^,^9^

A 2011 review of CAMHS services in 42 LMIC (Low and middle-income countries) found that less than 1% of beds in inpatient facilities are reserved for children and adolescents.^10^Placing the findings of foreign trend studies in Pakistani context is difficult because of the distinct absence of knowledge and research on the treatment of child and adolescent psychopathology in the country. However, some trends can be compared with few local studies of outpatient child psychiatry services. Most patients in this study requiring admission were females, unlike most outpatient service use by male youths reported in the same hospital setting in a previous study. 11This may be due to preponderance of dissociative disorder in our admitted population, a disorder more prevalent in adolescent girls in the local context. Median age of 13 years in the sample was in line with finding of a report of inpatient psychiatric care of children & adolescents in UK, that children 12 and older were more likely to be admitted for an acute inpatient stay than younger children.^12^The National In-patient Child and Adolescent Psychiatric Study (NICAPS) surveyed all Tier 4 CAMHS Units in England and Wales & found that majority of young children (<13 years) needing admission in National Health Service (NHS) were males whereas this proportion was higher for females in adolescent age group.^13^

Our clinical experience suggests severely disturbed behavior often with underlying intellectual disability as one of the most common reasons for admission of very young children in our facility. A significant proportion of patients were brought by family directly (55%), in contrast to developed countries like United Kingdom, where majority of referrals to Specialist inpatient services are through primary and secondary care services.^14^,^15^ The referral systems and development of community services in Pakistan is patchy and there is a lack of awareness among family physicians, school teachers and other medical specialties about pediatric psychiatric disorders, need for referrals and a more holistic approach to treatment. To address this issue, we have planned a series of outreach programs to educate and encourage school teachers and counsellors to identify and refer cases, and similarly establish a consultation-liaison relationship.

In our study, mean duration of admission was significantly less (almost two week) than reported from some studies from developed world as well as neighboring country India.^16^,^17^ The mean length of stay for cases discharged during the NICAPS study period was 115 days (3.7 months) ± 181 days (5.9 months) and a range of 0 to 2,194 days. Statistically significant differences between length of stay and diagnosis was observed.^13^ However, we do not think that our length of stay is particularly unusual, for example there is general decline in the length of stay (LOS) among mental health patients in the United States.^18^,^19^ Brief duration of admission in our local context is most likely explained by the inability of the parent to accompany his/her child during the hospital stay due to financial and household difficulties. Families preferred to be discharged and continue treatment / follow up in outpatient setting after symptomatic improvement of the patient. Inpatient environment also acts as a therapeutic intervention in certain situations; especially in dissociative and other stress related disorders.^20^ This study also highlights the diagnoses in inpatient population which has shown a slight divergence from general epidemiological trends. Significant proportion (41.32%) of young people requiring admission in this study were diagnosed as having Neurotic, Stress-related and Somatoform Disorders; Dissociative disorder being the largest subgroup. Similar trend was observed with dissociative disorders (which are less common in western settings), alongside speech problems, intellectual disability and behavior problems as the most common presentations in outpatients’ settings in Pakistan.^12^Vogel and Holford inpatient study reported the following main diagnostic categories: behavioral complaints (82%), (Attention deficit hyperactivity disorder) (70%), learning difficulties (48%), mood disorders (37%), abuse (31%), developmental disorders (25%), elimination disorders (17%) and psychoses (2%). Almost half of these patients in this study had not received any intervention or had any contact with social services prior to admission.^21^ This is because primary and secondary facilities at schools and clinics are not equipped to deal with early interventions, a scenario almost similar to that in Pakistan. Conduct disorder including conduct and emotional disorder, eating disorders and hyperkinetic disorders were the most common diagnosis in inpatient group (<13 years) in NICAPS study. Schizophrenia, delusional or psychotic disorders, eating disorders, Mood (affective) disorders were among the most common diagnoses in admitted youth >13 years old.^13^ Another UK study also suggested that children with mood disorder experienced the highest risk for admission to acute inpatient care. 12

The prevalence of somatization in children in Pakistan may be higher because children are less verbally expressive and may tend to selectively manifest physical symptoms as idioms of psychological distress. Presenting with physical complaints is much more acceptable culturally as compared to presenting with psychiatric symptoms. Evidence also suggest that activation of the immune system can induce somatization.^22^ These findings may be particularly relevant to children in developing countries like Pakistan who have higher exposure to infections and pathogens producing immunological challenges. A recent study also showed an association between medically unexplained symptoms with anxiety and depression in Pakistani children. Similar to earlier studies, higher percentage of the inpatient population (11.04%) also had comorbid ID in our study.^13^

While there are specialist units providing care and treatment to children and young people with a learning disability and co-morbid mental health problems in some of the developed countries, this is not the case in Pakistan and although not ideal by any means, but young people with ID and mental illness are either managed in general child & adolescent unit or adult psychiatric units depending on availability of beds in local services.^14^,^15^

The inpatient service provides care and treatment for children and adolescents who are experiencing mental disorders leading to significant impairment and/or risk. A higher percentage of the young in-patient population in England were also rated as having moderate to severe problems on all the HONOSCA scales.^13^ Diversity of disciplines in our CAMHS multidisciplinary team and various therapeutic interventions & holistic and comprehensive approach to pediatric psychiatric issues resulted in significant improvement in CGI (severity) scores from admission to discharge as well as correlated with CGI-improvement scores. Effective use of psychotropic medications led to very good efficacy index in the study. Pfeiffer and Strzelecki (1990) applied contemporary statistical methods to pooled data from 34 studies and concluded that there was fair evidence for benefit of inpatient admission, especially with careful planning of aftercare.^24^ Another study involving four inpatient units found general improvement, particularly in symptoms, following inpatient unit treatment.^25^ Specific improvement was seen in major affective disorders and schizophrenia while least improvement seen in conduct disorder in the same study. Advantages of use of SDQ and CGI tools are their brevity and ease of use, which makes them service friendly and helps in tracking the progress of patient over time

### Limitations of the study

The main limitation of inpatient service development in our unit was lack of classroom/ schooling facility because of lack of funding/ resources as children and young people in specialized CAMHS day/inpatient settings in developed countries generally participate in a structured program of education and therapeutic activities during their admission. Plans for incorporating structured education program for admitted youth in ward is currently underway. There are some caveats to the study findings. The present study of one center may not apply to other hospital settings. Information was restricted to diagnosis, and relevant demographic factors. We also did not address the details of treatment options, which would have been helpful too. It would be interesting to have qualitative data to understand experiences of families and children utilizing the inpatient services.

## CONCLUSIONS

We report on the successful establishment of a child and adolescent facility in a low-income country with no national child mental health policy. Although there is a need for enhanced service delivery in primary care and early intervention, we believe that there is a clear need for specialized services such as ours in low income countries. Our future plans include provision of formal caregivers’ groups, schooling within inpatient setting and promoting preventive aspect by working closely with schools. Limitations on funding and resources, staff-skill shortages, and inadequacies within sectors other than health (welfare, education, social services) are other pressing issues. In order to provide better delivery of children and adolescents, psychiatric services, in terms of both demand and supply, we need education of parents, children, institutions, physicians and providers at all levels of health care system to improve timely identification and referral of children and youth and effective evidence-based interventions.

### List of Abbreviations:

**ICD-10.** International Classification of Diseases-10.

**WHO**: World Health Organization.

**SDQ**: Strengths and difficulties questionnaire.

**CGI:** Clinical global Impression Scale.

**LOS**: length of stay.

**CAMHS:** Child & Adolescent Mental Health Services.

**LMIC:** Low and Middle-Income countries.

### Authors’ contributions:

**NI** conceived the study, literature review, participated in its design, coordination, analyzed the data and helped to draft the manuscript and also the responsible and accountable for the accuracy or integrity of the work.

**ZHB** helped in design, data collection, article drafting & critical revision.

**MWA** conceived the study, and helped in writing & reviewing the manuscript.

**RS** participated in design of the study, collected the data and helped in analysis.

**AA** helped in literature review & critical revision.

All authors read and approved the final manuscript.
